# Identifying the molecular mechanism of blood stasis syndrome through the symptom phenotype–genotype association approach: 

**DOI:** 10.1097/MD.0000000000040717

**Published:** 2024-12-06

**Authors:** Minh Nhat Tran, Hyeong Joon Jun, Sanghun Lee

**Affiliations:** a Korean Medicine Data Division, Korea Institute of Oriental Medicine, Daejeon, Republic of Korea; b College of Pharmacy, Chungnam National University, Daejeon, Republic of Korea; c Faculty of Traditional Medicine, Hue University of Medicine and Pharmacy, Hue University, Thua Thien Hue, Vietnam; d Korean Convergence Medical Science, University of Science and Technology, Daejeon, Republic of Korea.

**Keywords:** blood stasis syndrome, concept unified identifiers, molecular mechanism, phenotype–genotype association

## Abstract

In traditional medicine (TM), blood stasis syndrome (BSS) is characterized by insufficient blood flow, resulting in a group of symptoms such as fixed pain, a dark complexion, bleeding, and an astringent pulse. While BSS pathology has been previously explored, its molecular mechanisms remain elusive owing to challenges in linking TM symptoms to genes. Our study aimed to elucidate the mechanisms underlying BSS using a phenotype–genotype association approach. We extracted BSS symptoms from various studies, linked them to medical terms using a Unified Medical Language System, and connected these terms to genes in the DisGeNET database. The molecular network patterns of BSS symptoms were revealed through analyzing protein–protein interactions and symptom–gene associations. Our findings revealed 1325 associations between 16 BSS symptoms comprising 32 concept-unified identifier terms and 937 genes. Network analysis highlighted the centrality of JAK2, ITGB3, and F2, associated with multiple BSS symptoms (≥5 concept-unified identifier terms) and numerous protein interactions (≥20 interactions). Enrichment analysis indicated the involvement of BSS genes in the immune system (*P*-value = 4.49e‐14) and hemostasis (*P*-value = 1.28e‐07) pathways. BSS symptoms were linked to genes regulating blood coagulation, immune responses, blood flow, and inflammatory reactions. This approach may be extended to establish genotype networks for understanding TM pattern identifications, which are composed of diverse groups of symptoms, for personalized diagnosis and treatment.

## 1. Introduction

Pattern identification, also known as “bian zheng,” is a process in traditional medicine (TM) where clusters of symptoms are thoroughly examined to determine the location, category, and origin of syndromes, as well as the overall health of patients.^[1,2]^ Blood stasis syndrome (BSS) is one such syndrome within the pattern identification system, defined as a pathological phenomenon responsible for a specific cluster of symptoms, including fixed location pain, blood spots beneath the skin, hemorrhage, dark-purple face or tongue, and an astringent pulse.^[3]^ It is broadly referred to result from internal or external factors that render blood circulation insufficient or extremely poor. BSS holds significance in TM as it pertains to the blood, a fundamental element for nourishing and sustaining physiological functions in the body, which is involved in almost all cases of external trauma and chronic internal disorders.^[4]^ However, due to its nature as a collection of diverse symptoms, specifying the biological mechanisms underlying BSS has been challenging. Similar to other terms in TM, achieving a precise understanding of BSS from a modern biological perspective remains difficult.

Modern studies have investigated BSS. Asian experts have developed diagnostic tools for determining the incidence and presence of BSS in China,^[5]^ Japan,^[6]^ and Korea.^[7]^ Certain signs and symptoms have been established; however, a comparison of these tools revealed differences in the diagnostic criteria of BSS.^[8]^ BSS has been linked to distinct and specific conditions, including cancer, diabetes, stroke, and cardiovascular diseases; however, intercountry variations remain significant.^[8]^ For instance, Korean medicine practitioners have identified menstrual disorders and traumatic injuries as prevalent BSS-related conditions,^[9]^ whereas the Japanese literature on BSS suggests that it is also related to dermatological and gynecological diseases.^[10]^ The pathological features of BSS in biological rheology,^[10]^ thrombus formation,^[11]^ inflammation,^[12]^ accelerated senescence of red blood cells,^[13]^ and endothelium-derived vasoactive factors,^[14]^ have been established in both in vivo studies and clinical trials. Nonetheless, the biological mechanisms underlying BSS development remain unclear.

From a modern perspective, symptom phenotypes encompass signs and symptoms and stand out as the primary clinical features of diseases; they are essential for medical appointments, diagnosis, and treatment. Investigating the clinical patterns and biological processes associated with symptom phenotypes is believed to significantly enhance the field of precision medicine.^[15]^ Recent advances in phenotype–genotype associations and network biology have resulted in successful approaches to understanding the fundamental mechanisms of complicated phenotypes.^[16,17]^ These approaches are also useful for understanding the molecular mechanisms underlying TM patterns. An efficient method to link traditional symptom patterns with phenotypes and genotypes is required. SymMap was the first database to link numerous TM symptom–modern medicine symptom associations using manual curation by experts.^[18]^ Simultaneously, the development of medical vocabularies, such as the Systematized Nomenclature of Medicine Clinical Terms (SNOMED CT),^[19]^ Unified Medical Language System (UMLS),^[20]^ and Human Phenotype Ontology (HPO)^[21]^ has facilitated the unification and association of biomedical terms. For example, Shu et al linked 431 symptoms to 341 concept-unified identifier (CUI) codes and 3598 genes using UMLS to determine the diversity and molecular network patterns of symptom phenotypes.^[22]^ In addition, phenotype–genotype associations and network biology have been applied in TM to identify potential genes for certain symptoms,^[23]^ construct a phenotype–biology–herb network, or evaluate the non-specificity of symptom phenotypes.^[24]^ These approaches hold promise in overcoming the ambiguity of TM patterns, which are comprised of complex symptoms, making their biological mechanisms challenging to define. However, to date, no study has applied them to a specific pattern such as BSS.

**Figure 1.**
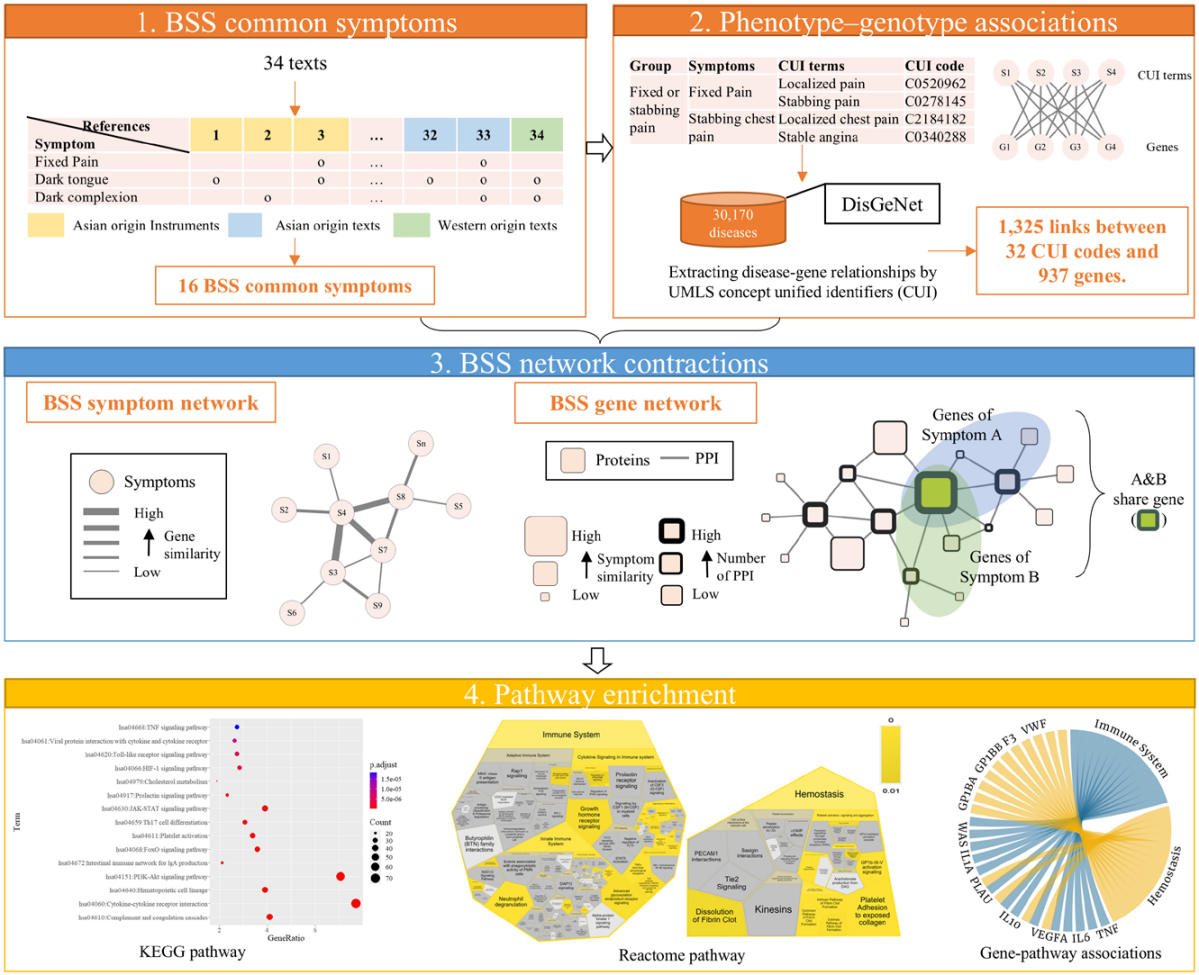
Work flow of the study.

Therefore, in this study, we employed a symptom phenotype–genotype association approach to explore the biological mechanisms involved in BSS. We first extracted common BSS symptoms from different texts based on a previous study and associated them with CUIs in UMLS to establish phenotype–genotype associations. Then, the symptom- and gene-related networks of the BSS were constructed to measure both the phenotypic and molecular importance of symptom phenotypes. Finally, we analyzed the pathways to elucidate the molecular mechanisms (Fig. 1). By linking BSS symptoms to genetic and molecular pathways, our findings offer evidence that can be validated through modern biomedical research, contributing to a clearer understanding of the ambiguity in pattern identification.

## 2. Methods

### 2.1. BSS clinical symptom manifestations from various studies

We curated the data related to the clinical symptoms of BSS based on our colleagues’ previous study on blood stasis.^[8]^ From this study, 34 texts related to blood stasis were collected, including 13 on diagnostic instruments,^[5–7,25–34]^ 21 on descriptions of BSS (03 WHO related publications,^[1,35,36]^ 15 textbooks and articles,^[37–51]^ 03 online educational sources^[52–54]^). Diagnostic tools were searched manually and electronically in the AMED, CENTRAL, EMBASE, and MEDLINE databases, while the remaining documents were manually searched only for English texts in personal and colleague collections, as well as school libraries.^[8]^

Symptoms associated with BSS were extracted and compared. We constructed tables with all the symptoms and signs associated with BSS to analyze the variety of manifestations. Due to the variation in the number of symptoms described in the 34 texts on BSS, only the common symptoms (those appearing in at least 4 texts) were selected for the next step of this study.

### 2.2. Symptom phenotype–genotype associations

Keywords related to the symptoms and symptom descriptions were then added into the UMLS Metathesaurus to search for relevant items in the CUI code for each concept. The UMLS is an extensive biomedical thesaurus structured by concepts or meanings, linking comparable names for the same concept from approximately 200 distinct vocabularies. The Metathesaurus also identifies meaningful relationships between concepts, preserving concept names, meanings, and relationships within each vocabulary.^[20]^

The vocabularies selected for the CUI code search in UMLS included ICD9, ICD10, DSM-5, SNOMEDCT, OMIM, MSH, NCBI, GO, HPO, MEDLINEPLUS, and WHO, which are vocabularies related to human species medicine. Substituting common TM terms with modern biomedical terms in UMLS is difficult. For example, the “Fixed pain” symptom is described as localized and stabbing pain, sensitive to pressure, but there is no CUI term for fixed pain in UMLS, so we choose CUI terms like “Localized pain” (C0520962), “Stabbing pain” (C0278145), and “Sensitive to touch” (C0423580) to describe the “Fixed pain” symptom. Therefore, to ensure accurate terminological mapping of genetic data for symptoms in BSS, we manually mapped TM terms to English terms of symptoms in UMLS. This mapping was conducted with the assistance of trained medical researchers in our author list who had more than 5 years of experience (e.g., MNT, HJJ, and SL). CUI terms that received more than 2 consent forms were used to collect the related genes.

The CUI codes were then imported into DisGeNET to collect phenotype–genotype associations. DisGeNET comprises 30,170 human phenotypes, traits, disorders, and diseases, with 21,671 genes and 1,134,942 gene-disease connections.^[55]^

### 2.3. Construction of the BSS network

Based on the phenotype–genotype associations, we constructed 2 networks related to BSS symptoms and genes. One network is the “BSS symptom network,” which addresses symptoms through CUI codes, while the other is the “BSS gene network,” which addresses BSS genes through protein interactions. Networks were visualized using Cytoscape software version 3.10.^[56]^

In the BSS symptom network, each CUI term (e.g., localized pain) was represented as a node, whereas associations between nodes were represented as edges. Node size corresponds to the number of genes linked to a specific CUI term, whereas nodes are interconnected if they share a common gene associated with both CUI terms. The edge size reflects the number of shared genes between 2 CUI terms.

In the BSS gene network, each gene is represented as a node, and the size of each node is represented by the number of CUIs associated with the gene. The larger the size, the more important the gene. Simultaneously, the links of these nodes are protein interactions obtained from the STRING database, which is a protein interaction-related database. In STRING, we selected only physical interactions, which are direct interactions confirmed through text mining, experiments, and databases.^[57]^ The Homo sapiens option for each species and the highest confidence level for the minimum required interaction score were set. The number of interactions for each gene was represented by the thickness of the node border associated with that gene. The thicker the border nodes, the greater the number of interacting genes.

### 2.4. Enrichment analysis

We employed enrichment analysis to identify the molecular pathways and interactions that may be affected by the BSS genes. Pathway analysis is a powerful tool for determining the biological roles of genes and proteins, allowing us to find significant pathways of overrepresented genes or proteins in a large collection that can be connected to specific disease characteristics. In this study, we obtained the functional enrichment analyses using the Database for Annotation, Visualization, and Integrated Discovery (DAVID, version 6.8)^[58]^ and Reactome.^[59]^ The adjusted *P*-value for each term, corrected using the false discovery rate method, was used to sort the data for the Kyoto Encyclopedia of Genes and Genomes (KEGG) and Reactome pathway enrichment. The KEGG PATHWAY database consists of organism-specific and manually generated reference pathway maps, whereas the Reactome consists of manually managed and peer-reviewed interactions, reactions, and pathways.

## 3. Results

### 3.1. BSS clinical symptom manifestations from different texts

From the 34 studies related to BSS, the symptoms and their descriptions were extracted from our previous study.^[8]^ We have rearranged similar descriptions of symptoms under broad headings to identify the common symptoms mentioned in the literature (Table S1, Supplemental Digital Content, http://links.lww.com/MD/O57). Table 1 lists the 16 signs/symptoms agreed upon in at least 4 studies (such as dark tongue, presence of masses, fixed pain, dark complexion, menstrual bleeding problems, and a choppy pulse), which were collected for association with the relevant genes in the next step.

**Table 1:** Frequency of symptoms in the different resources.

Sign/symptom	13 Asian origin instruments	13 Asian origin texts	8 Western origin texts	Total
Dark tongue	11	8	6	25
Masses	5	9	7	21
Fixed pain	5	8	7	20
Dark complexion	6	11	6	23
Menstrual bleeding problems	3	2	7	12
Choppy pulse (rough)	4	1	7	12
Dark lips (purple or blue lips)	8	7	6	21
Discolored (dark) spots on the tongue	6	6	3	15
Subcutaneous extravasation/ecchymoses	9	9	1	19
Dry skin	5	0	1	6
Darkened eyelids or around eyes	8	5	0	13
Spider nevi (fine capillaries/venules)	5	9	0	14
Darkened nails	1	3	5	9
Sublingual blood vessel changes	5	4	1	10
Dark stools	5	0	0	5
Stabbing chest pain	4	0	0	4

### 3.2. Phenotype–genotype associations

To obtain symptom–gene associations, we used the CUI code as an intermediary. The selection of CUI codes corresponding to TM symptoms was performed manually by 3 TM doctors with more than 5 years of clinical experience. Table 2 shows the CUI terms and CUI codes corresponding to BSS symptoms. Certain symptoms of similar nature, shown in Table 1, were rearranged into the same group, such as stabbing chest pain and fixed pain (all pain), and dark tongue, discolored spots on the tongue, and lingual blood vessel changes (all tongue expressions). Due to the specific nature of the symptoms, some symptoms related to the pulse and tongue could not be identified as related CUIs. The results showed that 52 CUI codes were associated with 16 TM symptoms of BSS.

**Table 2:** CUI symptom terms mapped into traditional symptom terms of BSS.

Group	Traditional symptoms	Related CUI terms in UMLS	CUI code
Fixed or stabbing pain	Fixed pain	Localized pain	C0520962
		Localized abdominal pain	C0522061
		Pain (sharp, boring, drilling, piercing)	C2675800
		Sharp pain	C0455270
		Stabbing pain	C0278145
		Sensitive to touch	C0423580
		Primary dysmenorrhea	C0149875
		Dysmenorrhea	C0013390
	Stabbing chest pain	Localized chest pain	C2184182
		Angina Pectoris	C0002962
		Angina, Unstable	C0002965
		Stable angina	C0340288
Dark tongue-related signs	Dark tongue	Blue tongue	C4284010
		Purple tongue discoloration (classic feature)	C3278100
		Hematoma of tongue	C0474983
	Discolored (dark) spots on the tongue		
	Sublingual blood vessel changes		
Dark face and nails	Dark complexion	Cyanosis of skin	C0010520
	Darkened eyelids or around eyes	Periorbital hyperpigmentation	C1844606
		Pigmentation of eyelids	C0854438
	Dark lips (Purple or blue lips)	Blue lips	C0240194
		Lip hyperpigmentation	C4021963
		Dark color of gums	C4280389
	Darkened nails		
Masses or lumps	Masses	Abdominal mass	C0000734
		Multiple masses	C1265602
		Masses soft tissue	C0457193
		Palpable mass	C0746412
		Abdominal or pelvic swelling, mass, or lump	C0476310
		Abdomen distended	C0000731
Choppy or rough pulse	Choppy pulse (rough)		
Organ bleeding	Menstrual bleeding problems	Irregular menstrual bleeding	C0156404
		Intermenstrual heavy bleeding	C0232943
		Hypomenorrhea	C0020624
		Menorrhagia	C0025323
		Thrombus	C0087086
	Dark stools	Melena	C0025222
		Hematochezia	C0018932
Subcutaneous extravasation/ecchymoses	Subcutaneous extravasation/ecchymoses (Red/purple speckles, maculae or petechiae)	Petechiae	C0031256
		Petechiae of skin	C0241144
		Maculae	C0332573
		Subcutaneous hemorrhage	C0854107
		Spots on skin	C0848332
		Internal hemorrhage	C1390214
		Muscle hematoma	C0240412
		Hematoma of skin	C0475852
		Subcutaneous hematoma	C0520532
		Subungual hematoma	C0474975
		Purpura	C0034150
		Ecchymosis	C0013491
	Spider nevi (fine capillaries/venules)	Spider nevus	C0085666
		Telangiectasia	C0039446
Dry skin	Dry skin	Dry skin	C0151908
		Rough skin	C0859038
		Scaly skin	C0423773
		Fissure in skin	C0221245

The CUI codes were then searched in DisGeNET, after removing the codes, for which related genes could not be found, 1325 associations involving 16 BSS symptoms, 32 CUI codes, and 937 genes were identified.

**Figure 2.**
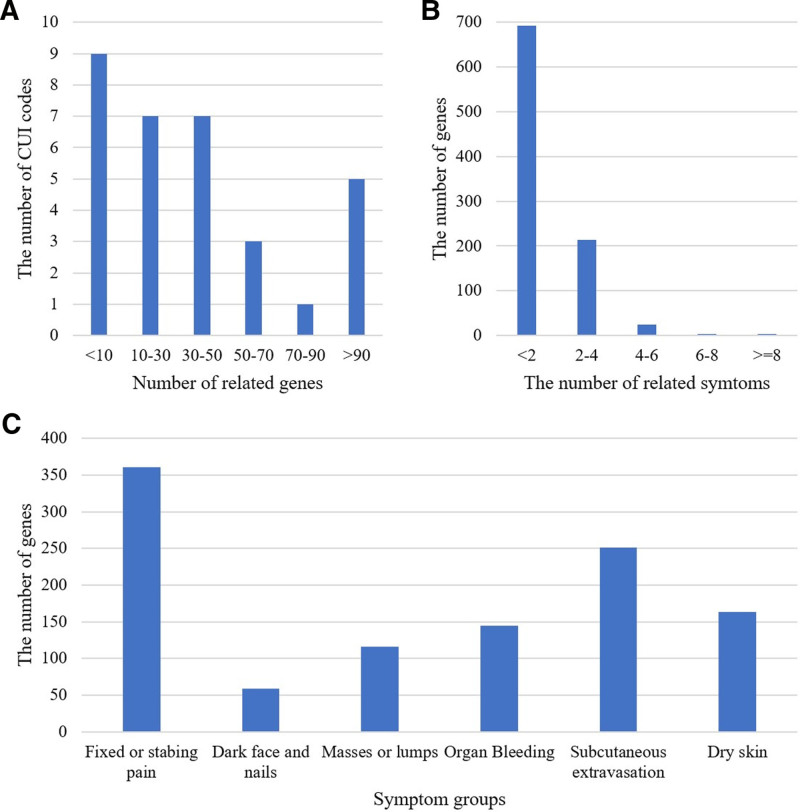
(A) Distribution of genes associated with symptoms. (B) Distribution of symptoms associated to genes. (C) Distribution of genes in symptom groups.

This analysis determined that an average of 41.41 related genes were found per CUI code; conversely, a single gene was associated with an average of 1.42 CUI codes. Particularly, 50% of the CUI codes had <30 associated genes (Fig. 2A); however, several CUI codes with more than 100 genes still existed, such as dry skin (159 genes), stable angina (144 genes), and distended abdomen (103 genes), suggesting an intricate underlying pathophysiology and potential comorbidities associated with these symptom phenotypes. Over 70% of the genes had one associated CUI code, whereas some genes, such as TNF, ITGA2B, GP1BA, PTEN, IL6, ITGB3, and CRP, were associated with more than 5 CUI codes (Fig. 2B). The symptom groups with the largest number of involved genes were “Fixed and stabbing pain” (361 genes), “subcutaneous extravasation” (251 genes), and “dry skin” (163 genes), as shown in Figure 2C.

### 3.3. Construction of the BSS symptom network

**Figure 3.**
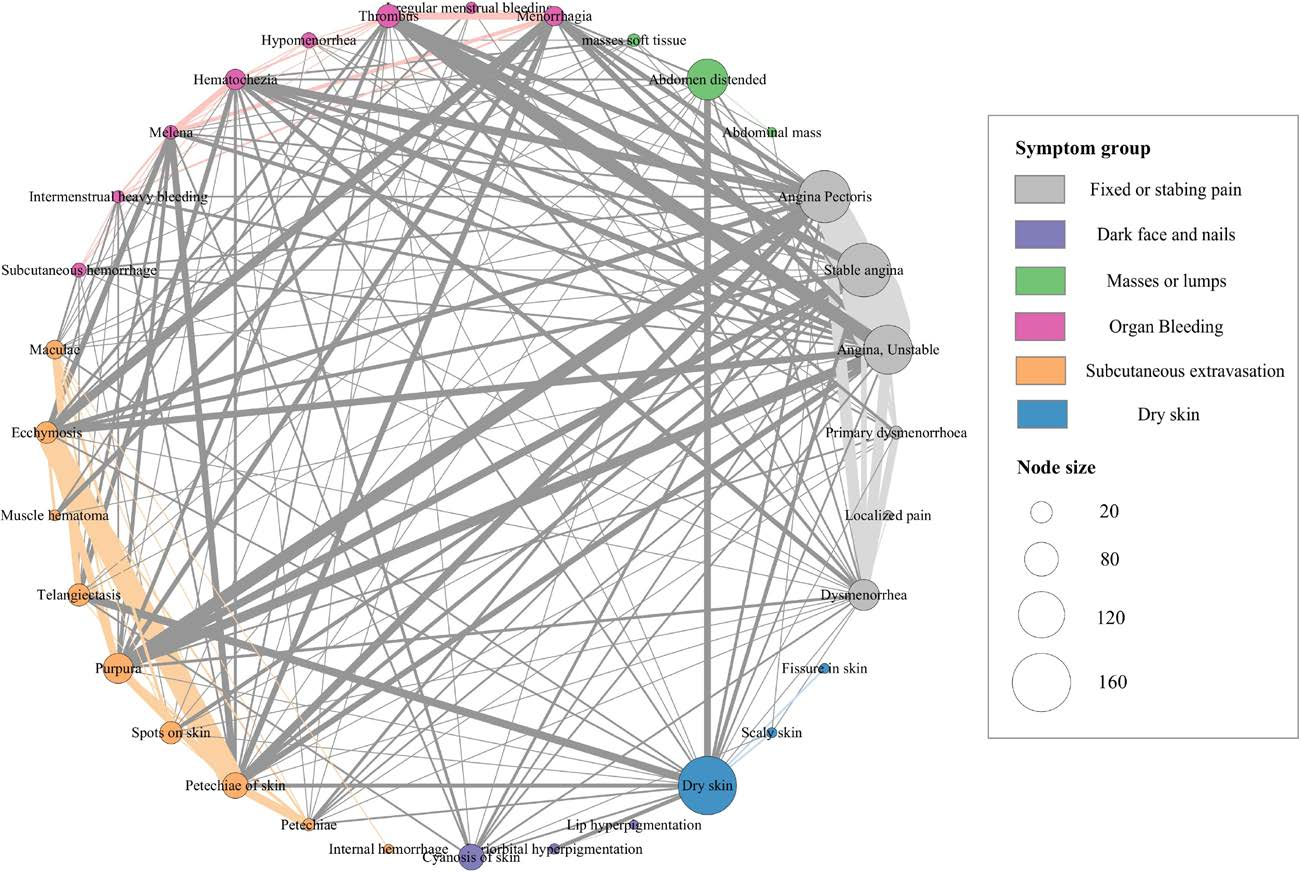
Blood stasis syndrome symptom network. Each node represents a distinct CUI term, which is color-coded based on its association with certain symptom groups, as indicated by the 6 groups. Linkages between CUI terms within the same symptom group are shaded with a dimmer color, whereas linkages joining other symptom groups are shown in gray. The node size is proportional to the number of genes linked with the appropriate CUI term, and the thickness of the linkages is proportional to the number of genes shared by the connected CUI terms. CUI = concept-unified identifier.

**Figure 4.**
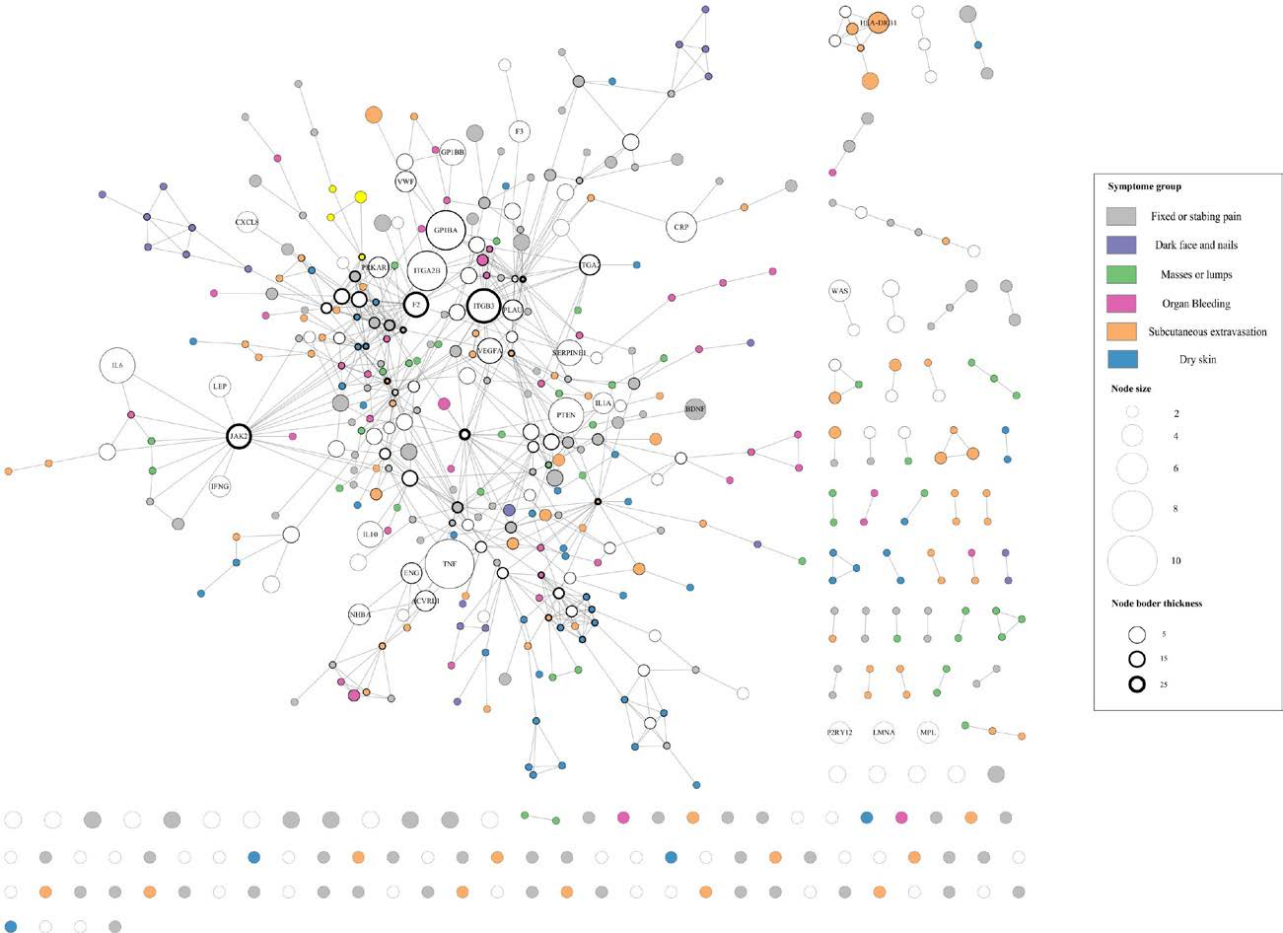
Blood stasis syndrome gene network. Nodes represent genes, and edges signify physical interactions between genes. The size of each node is determined by the number of CUI codes in which the gene is involved, while the thickness of each node corresponds to the number of connections around the node. Nodes appear in white if the associated genes are linked to more than one symptom group. Genes related to more than 4 CUI keywords and those mentioned in the text are indicated with their gene symbols. CUI = concept-unified identifier.

From the symptom–gene associations, we generated 2 biologically relevant networks (Figs. 3 and 4). In the “BSS symptom network,” nodes symbolize CUI terms, and 2 terms are connected if they share at least one associated gene associated (Fig. 3). In the “BSS gene network,” nodes represent genes, whereas connections between genes are established based on their physical interactions with each other (Fig. 4).

In the BSS symptom network, all CUI codes had at least one link to other codes, indicating that the genetic foundations of the majority were shared with other codes to a certain extent. The obtained BSS symptom network displayed several connections between individual symptoms and symptom groups (Fig. 3). Specifically, symptoms in the “Fixed and stabbing pain” group shared many genes, such as angina pectoris and dysmenorrhea (12 shared genes). The subcutaneous bleeding group was similar; for example, petechiae of the skin and ecchymosis (24 shared genes). In addition, groups such as pain, internal bleeding, and bleeding under the skin shared disease genes. However, the remaining groups, such as dry skin, masses or lumps, and dark faces or nails, showed limited connection with the other groups.

In the BSS gene network, the size of each node (representing a gene) is determined by the number of CUI codes in which the gene is implicated. Two genes are connected if they physically interact with each other, providing a gene-centric perspective of the disease. In this network, 423 of 937 BSS genes exhibited connections with other BSS genes, with 322 genes belonging to the giant component. Although the involvement of genes in multiple CUIs rapidly declined (Fig. 2B; white nodes in Fig. 4), several disease genes (e.g., TNF, ITGA2B, GP1BA, PTEN, IL6, ITGB3, and CRP) were involved in as many as 5 CUI codes, suggesting that these genes play a significant role in the pathogenesis of BSS. ITGB2, PTPN11, CTNNB1, JAK2, ITGB3, TP53, and F2 showed the highest number of protein interactions between genes. JAK2, ITGB3, and F2 are involved in many CUI codes (≥5 CUI terms) and protein interactions with other genes (≥20 interactions), representing major hubs in the network.

### 3.4. Enrichment analysis

**Figure 5.**
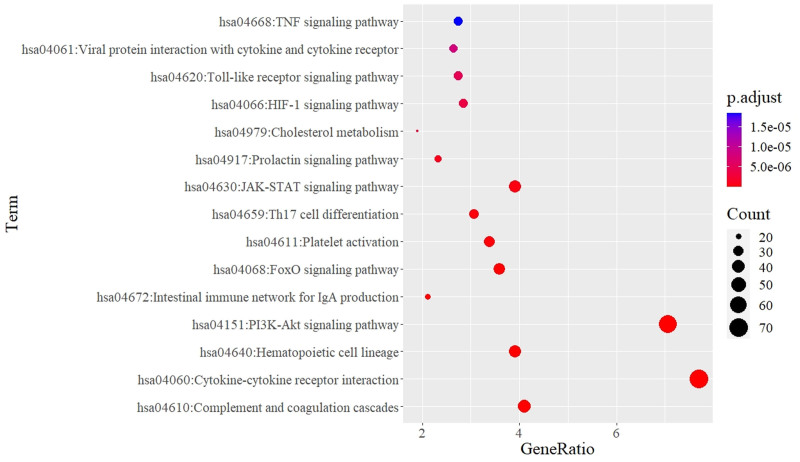
Kyoto Encyclopedia of Genes and Genomes pathway enrichment of blood stasis syndrome.

KEGG pathway enrichment analysis identified 102 signaling pathways (*P* < .01). The top 15 are shown in Figure 5. Pathways associated with specific diseases in the human disease category were removed to focus on other KEGG categories (e.g., organismal systems and environmental information processing) to provide more informative pathways explaining the molecular mechanisms of BSS. Many of the top 15 pathways are either related to the immune system, blood coagulation, or both, such as complement and coagulation cascades (hsa04610, *P*-value = 5.4e‐17), cytokine-cytokine receptor interaction (hsa04060, *P*-value = 9.2e‐16), hematopoietic cell lineage (hsa04640, *P*-value = 2.3e‐13), intestinal immune network for IgA production (hsa04640, *P*-value = 6.1e‐8), platelet activation (hsa04611, *P*-value = 1.9e‐7), Th17 cell differentiation (hsa04659: *P*-value = 3.6e‐7), and cholesterol metabolism (hsa04979, *P*-value = 2.4e‐6).

**Figure 6.**
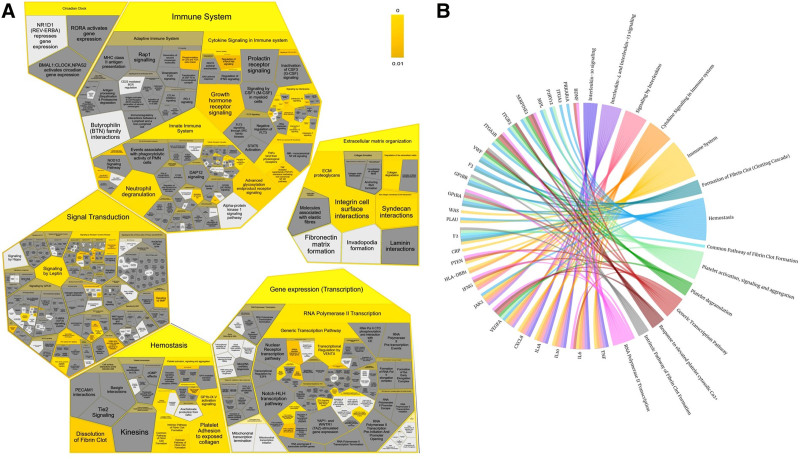
(A) Reactome pathway enrichment of blood stasis syndrome. (B) Involvement of key genes with the top Reactome pathways.

Reactome pathway enrichment analysis yielded 67 items (*P*-value < .01). The pathway groups and their sub-pathways at a significance threshold are displayed in Figure 6A. The pathway results were intensively enriched in the immune system, inflammation, and hemostasis (including IL-10, IL-4, and IL-13 signaling), formation of fibrin clots, platelet activation, signaling and aggregation, and platelet degranulation. Figure 6B shows the involvement of crucial genes (appearing in at least 4 CUI terms) with the top 15 Reactome pathways, such as TNF, IL6, IL10, IL10A, and CXCL8, involved in immune system-related pathways, whereas ITGB3, ITGA2, ITGA2B, F2, F3, GP1BA, GP1BB, and VWF were mainly involved in hemostasis. Genes such as JAK2, IFNG, and VEGFA were involved in both processes.

## 4. Discussion

Pattern identification is crucial in TM as it helps practitioners understand the underlying imbalances and disharmonies within the body. BSS is a common pattern characterized by stagnant blood circulation and poor microcirculation.^[60]^ Despite significant advancements in research on BSS, its precise molecular mechanisms remain unclear. The concept of blood stasis is different between TM and allopathic medicine. In allopathic medicine, it is often related to specific conditions that affect blood flow, such as blood clots, atherosclerosis, or venous insufficiency.^[61]^ In contrast, TM considers BSS to be a set of diverse symptoms that can manifest locally or affect the whole body, reflecting a broader imbalance in the body’s functions. The complexity of these symptoms makes exploring BSS difficult in TM. In this study, we employed the phenotype–genotype association approach to explore the molecular mechanisms of BSS by linking its symptoms to relevant genes. These results suggest that genes related to the BSS phenotype are involved in immune system pathways and hemostasis disorders, leading to blood flow disturbances and adverse immune responses.

Interpreting the mechanisms of TM patterns by linking symptoms to genotypes has been challenging. In this study, we extracted symptoms from BSS-related studies, used UMLS to find CUI codes, and linked them to CUI-code-related genes. As a result, 1325 associations were identified between 16 BSS symptoms, 32 CUI codes, and 937 genes. The BSS symptom network (Fig. 3) and the distribution of genes within symptom groups (Fig. 2c) revealed that symptom groups, such as fixed pain (mainly angina), organ bleeding (mainly menstrual disorder), and subcutaneous extravasation, were associated with many disease genes; these symptom groups shared common disease genes. This suggests that BSS is associated with several diseases in modern medicine, such as coronary heart disease,^[62]^ dysmenorrhea,^[63]^ and traumatic injury,^[64,65]^ rather than a specific disease.

Genes associated with multiple symptoms were identified in the BSS gene network, including TNF (10 CUI terms), ITGA2B, GP1BA (8 CUI terms), PTEN, IL6, and ITGB3 (7 CUI terms). Simultaneously, JAK2, ITGB3, F2, and ITGA2 were involved in multiple CUI codes and interacted with other genes (Fig. 4), suggesting that these are hub genes influencing blood stasis. ITGB3, ITGA2, and ITGA2B are integrins that are transmembrane receptors that promote cell-extracellular matrix and cell–cell adhesion. Integrins provide quick and flexible responses to signals at the cell surface (e.g., they signal platelets to initiate a reaction to coagulation factors). Integrin alpha-IIb/beta-3 (ITGA2B: ITGB3) is a receptor for vitronectin, thrombospondin, prothrombin, plasminogen, fibrinogen, and fibronectin. F2 (thrombin) and F3 (tissue factor) are key players in blood coagulation. Thrombin acts as an enzyme that converts fibrinogen into fibrin, thereby forming stable blood clots. It also activates coagulation factors and participates in cellular signaling.^[66]^ In contrast, tissue factors initiate coagulation by forming a complex with factor VIIa, leading to thrombin formation.^[67]^ In addition to their hemostatic roles, both proteins have non-hemostatic functions in inflammation, wound healing, and cell signaling pathways. GP1BA and GP1BB are essential platelet components that play crucial roles in platelet function and hemostasis. Under high shear conditions, GP1BA (glycoprotein Ib alpha) is involved in platelet binding and adhesion to von Willebrand factor (VWF). It forms a complex with GP1BB (glycoprotein Ib beta) and other subunits to create a glycoprotein Ib-IX-V receptor complex on the platelet surface. This complex facilitates platelet tethering and the initiation of platelet aggregation at locations of vascular damage.^[68]^ VWF is crucial in maintaining hemostasis by facilitating platelet adherence to vascular injury sites through the creation of a molecular bridge between the platelet–surface receptor complex, GPIb, and the subendothelial collagen matrix.^[69]^ Thus, most genes involved in the clotting process may develop coagulation disorders, leading to changes in blood flow. This was demonstrated by the gene enrichment results in KEGG and Reactome, where BSS genes participated in hemostasis pathways such as the formation of fibrin clots, platelet activation, signaling and aggregation, platelet degranulation, and complement and coagulation cascades (Figs. 5 and 6). A clinical study reported that individuals with BSS showed anomalies in platelet function^[70]^ and hemorheology.^[10]^

In addition, among the hub genes, many genes, such as TNF, IL6, IL10, CCLX8 (or IL8), and CRP were also involved in the immune system and regulation of inflammation. A strong association has been reported between BSS and inflammatory markers, such as CRP, IL6, TNF, and adhesion molecules. Stasis removal and blood circulation promotion are crucial in the clinical treatment of inflammation. Furthermore, experimental animal models of BSS have suggested that inflammatory reactions exhibit a certain level of mediation.^[71]^ Moreover, IL10 is an anti-inflammatory cytokine that limits immune responses and helps resolve inflammation. It downregulates the production of pro-inflammatory cytokines and inhibits immune cells.^[72]^ IL8 plays a role in neutrophil recruitment and activation during early inflammation.^[73]^ Together, TNF, IL6, CRP, IL8, and IL10 contribute to the complex balance between pro- and anti-inflammatory processes and play important roles in immune homeostasis and host defense mechanisms. In addition, JAK2, IFNG, and VEGFA contribute to the immune response and inflammation by regulating signaling pathways, modulating immune cell activation and function, and influencing the inflammatory microenvironment. Enrichment analysis showed that the BSS genes were involved in IL4, IL10, and IL13 signaling, cytokine signaling in the immune system, and Th17 cell differentiation. Therefore, the disease mechanism of BSS clearly involves disorders of pathways related to interleukins and cytokines in the immune system.

Our study had several limitations. The first is the inability to find CUI codes for TM-specific symptoms such as discolored spots on the tongue or choppy pulses. In TM, any disorder in the body leads to a change in the expression of the pulse and tongue; therefore, practitioners observe their abnormalities for diagnosis. This leads to unique characteristics in the dialect of TM; thus, identifying relevant CUI codes is difficult in modern medicine. Second, the number of linkages between CUI codes and genes was uneven, with many CUI codes involving only a few genes or no linkages. Once more, these codes mainly belong to symptoms such as dark tongue, stabbing pain, sensitivity to touch, and blue lips. Despite these challenges, we believe that the progress in symptom science will ultimately empower us to significantly broaden our data sources, thereby enhancing our understanding of symptom phenotypes in the post-genomic age.

In TM, accurate identification of diagnostic patterns is crucial for effective treatment. Clinical practitioners often rely on their experience and intuition to determine pattern identifications. By linking pattern identification symptoms to molecular mechanisms, this study provides additional evidence to complement traditional practices. The goal of this research is not to establish definitive correlations for immediate patient care, but to open a dialogue between traditional and modern medical approaches. Although the findings do not yet provide direct guidelines for patient care in TM, they lay the groundwork for future evidence-based approaches that could inform both traditional and modern medical fields.

In conclusion, our symptom phenotype-genotype association approach has shown that the molecular mechanisms involved in BSS symptoms are linked to coagulation and immune responses, aligning with the traditional medical concept of BSS. By connecting BSS symptoms to genetic and molecular pathways, this approach offers evidence that can be validated through modern biomedical research, helping to clarify the ambiguity in pattern identification. In the future, we aim to apply this method to other TM pattern identifications to build a comprehensive genotype network, thereby enhancing the efficacy of personalized diagnosis and treatment approaches.

## Author contributions

**Conceptualization:** Sanghun Lee, Minh Nhat Tran.

**Formal analysis:** Minh Nhat Tran.

**Methodology:** Minh Nhat Tran.

**Writing – original draft:** Minh Nhat Tran.

**Writing – review & editing:** Hyeong Joon Jun, Sanghun Lee.

## Supplementary Material


