# Supplementary material for: Identifying the molecular mechanism of blood stasis syndrome through the symptom phenotype–genotype association approach:
Source: Medicine (Baltimore). 2024 Dec 6;103(49):e40717. doi: 10.1097/MD.0000000000040717 (PMC11631032; doi:10.1097/MD.0000000000040717)
Supplement: Supplementary file 1 [file medi-103-e40717-s001.docx]

**Table S1.** Symptoms of blood stasis syndrome and their description in the texts.

| **Items** | **References** | | | | | | | | | | | | | | | | | | | | | | | | | | | | | | | | | |
| --- | --- | --- | --- | --- | --- | --- | --- | --- | --- | --- | --- | --- | --- | --- | --- | --- | --- | --- | --- | --- | --- | --- | --- | --- | --- | --- | --- | --- | --- | --- | --- | --- | --- | --- |
|  | 1 | 2 | 3 | 4 | 5 | 6 | 7 | 8 | 9 | 10 | 11 | 12 | 13 | 14 | 15 | 16 | 17 | 18 | 19 | 20 | 21 | 22 | 23 | 24 | 25 | 26 | 27 | 28 | 29 | 30 | 31 | 32 | 33 | 34 |
| **Fixed Pain** |  |  |  |  |  |  |  |  |  |  |  |  |  |  |  |  |  |  |  |  |  |  |  |  |  |  |  |  |  |  |  |  |  |  |
| Pain – fixed/localized stabbing pain (boring) (severe) (piercing) |  |  | 🗸 | 🗸 | 🗸 | 🗸 | 🗸 |  |  |  |  | 🗸 |  | 🗸 |  | 🗸 | 🗸 |  | 🗸 | 🗸 |  |  |  |  |  |  |  |  |  |  |  |  |  |  |
| Fixed pain / (Fixed location, confined to locality of obstruction. Stabbing pain is rare) |  |  |  |  |  |  |  | 🗸 |  |  | 🗸 |  |  |  |  |  |  |  |  |  |  |  |  |  |  |  |  |  |  |  |  |  |  |  |
| General fixed pain (or also stabbing, sensitive to pressure and chronic) |  |  |  |  |  |  |  |  | 🗸 |  |  |  |  |  |  |  |  |  |  |  |  |  | 🗸 |  |  |  |  |  |  | 🗸 |  |  |  |  |
| Fixed stabbing pain, aggravated with pressure/at night, better by day |  |  |  |  |  |  |  |  |  |  |  |  |  |  |  |  |  | 🗸 |  |  |  |  |  |  |  |  |  |  | 🗸 |  |  |  | 🗸 |  |
| Fixed pain in limbs |  |  |  |  |  |  |  |  |  |  |  |  |  |  |  |  |  |  |  |  |  |  |  |  |  | 🗸 |  |  |  |  |  |  |  |  |
| **Dark tongue** |  |  |  |  |  |  |  |  |  |  |  |  |  |  |  |  |  |  |  |  |  |  |  |  |  |  |  |  |  |  |  |  |  |  |
| (Dark) purple tongue / (dark red tongue) (red purple, blue purple or purple with spots) / dark purple around edge tongue | 🗸 |  |  |  |  | 🗸 |  |  |  | 🗸 |  |  |  |  |  | 🗸 | 🗸 |  | 🗸 | 🗸 | 🗸 | 🗸 | 🗸 | 🗸 |  | 🗸 |  | 🗸 | 🗸 |  | 🗸 | 🗸 | 🗸 | 🗸 |
| Tongue dark, purple with stasis speckles (purple or spotted tongue), (dark purple with ecchymoses/petechiae) |  |  | 🗸 |  |  |  |  | 🗸 | 🗸 |  | 🗸 |  |  | 🗸 |  |  |  | 🗸 |  |  |  |  |  |  |  |  |  |  |  |  |  |  |  |  |
| Bluish, purple tongue, (cyanotic tongue) |  |  |  |  |  |  |  |  |  |  |  |  |  |  |  |  |  |  |  |  |  |  |  |  |  |  | 🗸 |  |  |  |  |  |  | 🗸 |
| **Dark complexion** |  |  |  |  |  |  |  |  |  |  |  |  |  |  |  |  |  |  |  |  |  |  |  |  |  |  |  |  |  |  |  |  |  |  |
| Dark complexion / (soot-black complexion), (dusky face) |  |  | 🗸 |  |  | 🗸 |  | 🗸 |  | 🗸 | 🗸 | 🗸 | 🗸 | 🗸 |  | 🗸 | 🗸 | 🗸 | 🗸 | 🗸 | 🗸 |  |  | 🗸 |  |  | 🗸 |  | 🗸 |  |  |  | 🗸 | 🗸 |
| Somber facial complexion |  |  |  |  |  |  |  |  |  |  |  |  |  |  |  |  |  |  |  |  |  |  |  |  |  | 🗸 |  |  |  |  |  |  |  |  |
| **Masses** |  |  |  |  |  |  |  |  |  |  |  |  |  |  |  |  |  |  |  |  |  |  |  |  |  |  |  |  |  |  |  |  |  |  |
| Masses and swellings / tumors, cysts or swellings/lumps /masses |  |  | 🗸 |  | 🗸 |  |  |  |  | 🗸 |  | 🗸 |  | 🗸 | 🗸 |  |  |  |  |  |  |  |  |  |  |  |  |  |  |  |  |  |  |  |
| Palpable abdominal masses (resistances) |  |  |  |  |  |  |  |  | 🗸 |  |  |  |  |  |  |  |  |  |  |  |  |  |  |  |  |  |  |  |  |  |  |  |  |  |
| Visible painful and tender purple mass / (local bluish and purplish lump) / (Cyan-purple swellings (bruises) [e.g., from impact trauma]) | 🗸 | 🗸 |  |  |  |  |  | 🗸 |  |  | 🗸 |  |  |  |  |  |  |  |  |  |  |  |  |  |  |  |  |  |  |  |  |  |  |  |
| Swelling: generally fixed. Local bruise in the body surface or hard and palpable abdominal mass inside the body cavity can occur. |  |  |  |  |  |  |  |  |  |  |  |  |  |  |  |  |  | 🗸 |  |  |  |  |  |  |  |  |  |  |  |  |  |  |  |  |
| Abdominal mass with stabbing pain and tenderness | 🗸 | 🗸 |  |  |  |  |  |  |  |  |  |  |  |  |  |  |  |  |  |  |  |  |  |  |  |  |  |  |  |  |  |  |  |  |
| Abdominal masses that do not move |  |  |  |  |  | 🗸 |  |  |  |  |  |  |  | 🗸 |  |  | 🗸 |  |  |  |  |  |  |  |  |  |  |  |  |  |  |  |  |  |
| Abdominal accumulations (inflammatory or non-inflammatory masses, tumors, or cancers) |  |  |  |  |  |  |  |  |  |  |  |  |  |  |  | 🗸 |  |  |  |  |  |  |  | 🗸 |  |  |  |  |  |  |  | 🗸 |  | 🗸 |
| Fixed mass tumors, firm on palpation / (Hard localized tumors or masses) / (Internally- relatively hard swellings that can develop into concretions and accumulations) |  |  |  | 🗸 |  |  | 🗸 |  |  |  | 🗸 |  |  |  |  |  |  |  |  | 🗸 |  |  |  |  |  |  |  |  |  |  |  |  |  |  |
| Immovable mass in right upper quadrant |  |  |  |  |  |  |  |  |  |  |  |  |  |  |  |  |  |  |  |  |  |  |  |  |  |  |  |  | 🗸 |  |  |  |  |  |
| Pathogenic lump |  |  |  |  |  |  |  |  |  |  |  |  |  |  |  |  |  |  |  |  |  |  | 🗸 |  |  |  |  |  |  |  |  |  |  |  |
| **Dark lips (Purple or blue lips)** |  |  |  |  |  |  |  |  |  |  |  |  |  |  |  |  |  |  |  |  |  |  |  |  |  |  |  |  |  |  |  |  |  |  |
| Dark (purple/ black /red) lips / Violet oral lips and onyx (cyanotic lips) / blue lips |  |  |  |  |  | 🗸 | 🗸 | 🗸 |  |  |  |  |  |  |  |  | 🗸 | 🗸 | 🗸 | 🗸 | 🗸 | 🗸 |  | 🗸 | 🗸 | 🗸 | 🗸 | 🗸 |  |  |  | 🗸 |  | 🗸 |
| Lips, tongue or gums dark reddish or purple |  |  |  |  |  |  |  |  |  |  |  |  |  |  |  |  |  |  |  |  | 🗸 |  |  |  |  |  |  |  |  |  |  |  |  |  |
| **Darkened nails** |  |  |  |  |  |  |  |  |  |  |  |  |  |  |  |  |  |  |  |  |  |  |  |  |  |  |  |  |  |  |  |  |  |  |
| Purple nails / purple nails, edge nail root dark red |  |  |  |  |  | 🗸 |  |  |  |  |  |  |  |  |  |  | 🗸 | 🗸 | 🗸 | 🗸 | 🗸 |  |  |  |  | 🗸 |  |  |  |  |  |  |  |  |
| **Choppy pulse (rough)** |  |  |  |  |  |  |  |  |  |  |  |  |  |  |  |  |  |  |  |  |  |  |  |  |  |  |  |  |  |  |  |  |  |  |
| Fine choppy or irregular pulse/choppy, knotted or intermittent pulse | 🗸 |  |  |  |  |  |  |  |  |  |  |  |  |  |  |  |  | 🗸 |  |  |  |  |  |  |  |  |  |  |  |  |  |  |  |  |
| Pulse fine and rough (choppy) / choppy pulse |  |  | 🗸 |  |  |  |  |  |  | 🗸 | 🗸 |  |  | 🗸 |  | 🗸 |  |  |  |  |  |  |  |  |  |  |  |  |  |  |  |  |  |  |
| Wiry, firm or choppy pulse (wiry or choppy pulse) |  |  |  |  |  | 🗸 |  | 🗸 |  |  |  |  |  |  |  |  | 🗸 |  | 🗸 | 🗸 |  |  |  |  |  |  |  |  |  |  |  |  |  |  |
| Pulse string-like (wiry) and rough |  |  |  |  |  |  | 🗸 |  |  |  |  |  |  |  |  |  |  |  |  |  |  |  |  |  |  |  |  |  |  |  |  |  |  |  |
| Typical rough pulse or no pulse |  |  |  |  |  |  |  |  | 🗸 |  |  |  |  |  |  |  |  |  |  |  |  |  |  |  |  |  |  |  |  |  |  |  |  |  |
| Rough pulse or bound and intermittent pulse |  |  |  |  |  |  |  |  |  |  |  |  |  |  |  |  |  |  |  |  |  |  |  |  |  | 🗸 |  |  |  |  |  |  |  |  |
| Rough pulse |  |  |  |  |  |  |  |  |  |  |  |  |  |  |  |  |  |  |  |  |  |  |  | 🗸 |  |  | 🗸 |  |  |  |  |  |  | 🗸 |
| **Discolored (dark) spots on the tongue** |  |  |  |  |  |  |  |  |  |  |  |  |  |  |  |  |  |  |  |  |  |  |  |  |  |  |  |  |  |  |  |  |  |  |
| spotted tongue, dark purple with ecchymoses/petechiae |  |  | 🗸 |  |  |  |  | 🗸 | 🗸 |  | 🗸 |  |  | 🗸 |  |  |  | 🗸 | 🗸 |  |  |  |  |  |  |  |  |  |  | 🗸 |  |  |  |  |
| Stasis speckle or macule on tongue / (Purple spots / petechiae on the tongue) (red, white or black spots) |  |  |  | 🗸 | 🗸 |  |  |  |  |  |  |  |  |  |  |  |  |  |  |  |  |  |  |  |  | 🗸 | 🗸 | 🗸 |  |  |  |  | 🗸 | 🗸 |
| **Menstrual bleeding problems** |  |  |  |  |  |  |  |  |  |  |  |  |  |  |  |  |  |  |  |  |  |  |  |  |  |  |  |  |  |  |  |  |  |  |
| Bleeding of dark (purple) blood with (dark) clots | 🗸 |  | 🗸 | 🗸 | 🗸 | 🗸 | 🗸 | 🗸 |  |  | 🗸 | 🗸 |  | 🗸 |  |  | 🗸 |  | 🗸 | 🗸 |  |  |  |  |  |  |  |  |  |  |  |  |  |  |
| Hemorrhaging (especially post-partum or with irregular menses) |  |  |  |  |  |  |  |  |  | 🗸 |  | 🗸 |  | 🗸 |  |  |  |  |  |  |  |  |  |  |  |  |  |  |  |  |  |  |  |  |
| Bleeding: generally manifested as scanty in quantity with unsmooth flow, purplish in color with clots. |  |  |  |  |  |  |  |  |  |  |  |  |  |  |  |  |  | 🗸 |  |  |  |  |  |  |  |  |  |  |  |  |  |  |  |  |
| Pain in the lower abdomen during menstruation, scant menstruation with blood clots |  |  |  |  |  |  |  |  |  |  |  |  |  |  |  | 🗸 |  |  |  |  |  |  |  |  |  |  |  |  |  |  |  |  |  |  |
| Paroxysmal hemorrhage, especially in menstrual irregularities and postpartum disorders |  |  |  |  |  |  |  |  |  |  | 🗸 |  |  |  |  |  |  |  |  |  |  |  |  |  |  |  |  |  |  |  |  |  |  |  |
| Dark menstrual blood with clots / (dark lumps in menstrual blood and/or dark menstrual blood) |  |  |  |  |  |  |  |  |  |  |  |  |  |  |  |  |  |  |  |  |  |  |  |  | 🗸 | 🗸 |  |  |  |  |  |  | 🗸 |  |
| **Sublingual blood vessel changes** |  |  |  |  |  |  |  |  |  |  |  |  |  |  |  |  |  |  |  |  |  |  |  |  |  |  |  |  |  |  |  |  |  |  |
| Congested sublingual veins |  |  |  |  |  |  | 🗸 |  |  |  |  |  |  |  |  |  |  |  |  |  |  |  |  |  |  |  |  |  |  |  |  |  |  |  |
| Stagnation, varicosity, bruising of sublingual blood vessels / (tongue varicose veins), (sublingual varices) |  |  |  |  |  |  |  |  |  |  |  |  |  |  |  |  |  |  |  |  |  |  | 🗸 | 🗸 |  | 🗸 |  | 🗸 |  | 🗸 |  |  |  |  |
| **Subcutaneous extravasation/ecchymoses (Red/purple speckles, maculae or petechiae)** |  |  |  |  |  |  |  |  |  |  |  |  |  |  |  |  |  |  |  |  |  |  |  |  |  |  |  |  |  |  |  |  |  |  |
| Red speckles, purple macules, petechiae |  |  | 🗸 | 🗸 | 🗸 |  | 🗸 |  |  |  |  | 🗸 | 🗸 |  |  |  |  |  |  |  |  |  |  |  |  |  |  |  |  |  |  |  |  |  |
| Skin bruises easily and ecchymosis / /bruise easily |  |  |  |  |  |  |  |  |  |  |  |  |  |  |  |  |  |  |  |  |  |  |  | 🗸 | 🗸 |  |  |  |  |  |  | 🗸 |  |  |
| Subcutaneous extravasation/hypodermal ecchymoses |  |  |  |  |  |  |  |  |  |  |  |  |  |  |  |  |  |  |  |  |  | 🗸 | 🗸 | 🗸 |  |  |  |  | 🗸 |  |  |  |  |  |
| Purpura in derma (purple macula) |  |  |  |  |  |  |  |  |  |  |  |  |  |  |  |  |  |  |  |  |  |  |  |  |  |  | 🗸 |  |  |  |  |  | 🗸 | 🗸 |
| **Dry skin** |  |  |  |  |  |  |  |  |  |  |  |  |  |  |  |  |  |  |  |  |  |  |  |  |  |  |  |  |  |  |  |  |  |  |
| Skin-rough, dry, lusterless (squamous and dry skin) |  |  | 🗸 |  |  |  |  |  |  |  | 🗸 |  | 🗸 |  |  |  |  | 🗸 |  |  | 🗸 |  |  |  |  |  |  |  |  |  |  |  |  |  |
| Dry, scaly or cracked skin |  |  |  |  |  |  |  |  | 🗸 | 🗸 |  | 🗸 |  |  |  | 🗸 |  |  |  |  |  |  |  |  |  |  |  |  |  |  |  |  |  |  |
| Dermal roughness and chapping (rough skin) / (scaly skin), (dry skin) |  |  |  |  |  |  |  |  |  |  |  |  |  |  |  |  |  |  |  |  |  | 🗸 | 🗸 |  |  |  |  | 🗸 | 🗸 |  |  |  | 🗸 |  |
| **Darkened eyelids or around eyes** |  |  |  |  |  |  |  |  |  |  |  |  |  |  |  |  |  |  |  |  |  |  |  |  |  |  |  |  |  |  |  |  |  |  |
| Dark circles around your eyes |  |  |  |  |  |  |  |  |  | 🗸 |  | 🗸 |  |  |  |  |  |  |  |  | 🗸 |  |  | 🗸 | 🗸 |  | 🗸 |  |  |  |  | 🗸 |  |  |
| Pigmentation (darkening/black) of eyelids |  |  |  |  |  |  |  |  |  |  |  |  |  |  |  |  |  |  |  |  |  | 🗸 |  |  |  | 🗸 |  | 🗸 |  |  |  |  |  | 🗸 |
| **Spider nevi (fine capillaries/venules)** |  |  |  |  |  |  |  |  |  |  |  |  |  |  |  |  |  |  |  |  |  |  |  |  |  |  |  |  |  |  |  |  |  |  |
| Blood vessel changes (curved, thicker) and blood stasis signs on the skin (petechiae, spider naevi, subcutaneous dark blue spots etc.) |  |  |  |  |  |  |  |  | 🗸 |  |  |  | 🗸 |  |  |  |  |  |  |  |  |  |  |  |  |  |  |  |  |  |  |  |  |  |
| Spider nevi |  |  | 🗸 |  |  |  |  |  |  |  | 🗸 |  | 🗸 |  |  |  |  |  |  |  |  |  |  |  |  |  |  |  |  |  |  |  |  |  |
| Fine arteries - dilated venules, spider nevi / (Telangiectasis/vascular spiders) / (superficial capillary) |  |  |  |  |  |  |  |  |  |  |  |  |  |  |  |  |  |  |  |  |  | 🗸 | 🗸 | 🗸 |  |  |  |  | 🗸 |  |  |  |  |  |
| Fine capillary blood vessels on cheekbones |  |  |  |  |  |  |  |  |  |  |  |  |  |  |  |  |  |  |  |  |  |  |  |  | 🗸 |  |  |  |  |  |  |  |  |  |
| **Dark stools** |  |  |  |  |  |  |  |  |  |  |  |  |  |  |  |  |  |  |  |  |  |  |  |  |  |  |  |  |  |  |  |  |  |  |
| Dark stool (melena) / black stool |  |  |  |  |  |  |  |  |  |  |  |  |  |  |  |  |  |  |  |  |  |  | 🗸 | 🗸 |  |  |  |  |  |  | 🗸 |  |  |  |
| **Stabbing chest pain** |  |  |  |  |  |  |  |  |  |  |  |  |  |  |  |  |  |  |  |  |  |  |  |  |  |  |  |  |  |  |  |  |  |  |
| Stabbing chest pain / (angina / persistent angina) (chest pain) |  |  |  |  |  |  |  |  |  |  |  |  |  |  |  |  |  |  |  |  |  |  | 🗸 | 🗸 |  |  | 🗸 | 🗸 |  |  |  |  |  |  |

1= WHO 2007 [1]; 2= Li 2010 [2]; 3= Wiseman, Feng 1997 [3]; 4 = Cheng 1987 [4]; 5= Beijing College of Traditional Chinese Medicine 1980 [5]; 6= Maciocia 1989 [6]; 7= Hecker et al. 2005 [7]; 8= ICD-11 2017 [8]; 9= Neeb 2007 [9]; 10= Liu 1988 [10]; 11= Wiseman Ellis 1985 [11]; 12=Tsay 1995 [12]; 13= Manaka et al. 1995 [13]; 14= Kaptchuk 2000 [14]; 15= Beinfield, Korngold 1991 [15]; 16= Chen 2004 [16]; 17= Maciocia 2015 [17]; 18= TCMWiki 2017 [18]; 19= Sacredlotus 2017 [19]; 20= Yinyang house 2016 [20]; 21= Tsuneo 2010 [21]; 22 = Terasawa 1989 [22]; 23 = Li et al. 2014 [23];24= Lee et al. 2015 [24]; 25 = Su et al. 2013 [25]; 26 = Cheng et al. 2013 [26]; 27 = Kang et al. 2012 [27]; 28= Yao et al. 2009 [28]; 29= Guan, He 2014 [29]; 30= Tang et al. 2012 [30]; 31= Jung et al. 2016 [31]; 32= Park et al. 2013 [32]; 33 = Han et al. 2016 [33]; 34 = Lee et al. 2012 [34].

**References**

1 World Health Organization. Regional Office for the Western Pacific. *WHO international standard terminologies on traditional medicine in the Western Pacific Region*. (WHO Regional Office for the Western Pacific, 2007).

2 Li, Z.-G. & Pan, S.-L. Comparative study on WHO Western Pacific Region and World Federation of Chinese Medicine Societies international standard terminologies on traditional medicine: an analysis of the Five Sensory Organs. *J. Integr. Med.* **7**, 183-186 (2009). <https://doi.org/10.3736/jcim20090218>

3 Wiseman, N. *A practical dictionary of Chinese medicine*. (Paradigm Pubns, 1998).

4 Cheng, X. & Deng, L. *Chinese acupuncture and moxibustion*. (Foreign Language Press Beijing, 1999).

5 Beijing Zhong yi xue yuan. *Essentials of Chinese acupuncture*. (Elsevier Science & Technology, 1980).

6 Maciocia, G. *The foundations of Chinese medicine*. (Churchill Livingstone, 1989).

7 Hecker, H.-U., Steveling, A., Peuker, E. T. & Kastner, J. *Practice of Acupuncture: Point Location-Treatment Options-TCM Basics*. (Georg Thieme Verlag, 2004).

8 *ICD-11 codes*, <<http://apps.who.int/classifications/icd11/browse/l-m/en#/http%3a%2f%2fid.who.int%2fictm%2fentity%2f256171555>> (2017).

9 Neeb, G. R. *Blood stasis: China's classical concept in modern medicine*. (Elsevier Health Sciences, 2007).

10 Liu, Y., Vian, K. & Eckman, P. *The essential book of traditional chinese medicine: clinical practice*. Vol. 2 (Columbia University Press, 1988).

11 Wiseman, N. & Ellis, A. Translation of Zhong Yi Xue Ji Chu, 1975 as: Fundamentals of Chinese Medicine. *Brookline (USA): Paradigm Publications* (1985).

12 Tsay, K. Acupuncturist's Handbook. *Chestnut Hill, MA: CPM Whole Health* (1995).

13 Manaka, Y., Itaya, K. & Birch, S. *Chasing the dragon's tail: the theory and practice of acupuncture in the work of Yoshio Manaka*. (Paradigm Publications, 1995).

14 Kaptchuk, T. J. *Chinese medicine: the web that has no weaver*. (Random House, 2000).

15 Beinfield, H. & Korngold, E. *Between heaven and earth: A guide to Chinese medicine*. (Ballantine Books, 1991).

16 Chen, P. *Diagnosis in traditional Chinese medicine*. (Paradigm Publications, 2004).

17 Maciocia, G. *The Foundations of Chinese Medicine: A Comprehensive Text*. (Churchill Livingstone, 2015).

18 TCMWiki. *Stagnant blood*, <<https://tcmwiki.com/wiki/stagnant-blood>> (2017).

19 SacredLotus. *Blood stagnation*, <<https://sacredlotus.com/go/diagnosis-chinese-medicine/get/differentiation-syndromes-qi-blood-fluids-tcm>> (2017).

20 House, Y. *TCM Diagnostic Patterns - Qi Deficiency, Blood Stagnation*, <<https://theory.yinyanghouse.com/theory/chinese/tcm_diagnosis_meanings>> (2016).

21 Hisazumi, T. Oketsu-sho: Blood Stagnation Syndrome **17**, 14-15 (2010).

22 Terasawa, K. The presentation of diagnostic criteria for "Yu-xie"(stagnated blood) conformation. *Int. J. Orient. Med.* **14**, 194-213 (1989).

23 Li, S.-M., Xu, H. & Chen, K.-J. The diagnostic criteria of blood-stasis syndrome: Considerations for standardization of pattern identification. *Chin. J. Integr. Med.* **20**, 483-489 (2014). <https://doi.org/10.1007/s11655-014-1803-9>

24 Lee, J. A. *et al.* CORE-DITEC-BS (COnvergence REsearch of the DIagnostic TEChnology for Blood Stasis): Study protocol. *Eur. J. Integr. Med.* **7**, 417-422 (2015). <https://doi.org/10.1016/j.eujim.2014.10.009>

25 Shan-Yu, S., Chung-Hsien, Y., Chuang-Chien, C. & Qi, W. Acoustic Features for Identifying Constitutions in Traditional Chinese Medicine. *J. Altern. Complement. Med.* **19**, 569-576 (2013). <https://doi.org/10.1089/acm.2012.0478>

26 Shu-Chen, C. *et al.* Fire-Heat and Qi Deficiency Syndromes as Predictors of Short-term Prognosis of Acute Ischemic Stroke. *J. Altern. Complement. Med.* **19**, 721-728 (2013). <https://doi.org/10.1089/acm.2012.0546>

27 Kang, B.-K. *et al.* Reliability and validity of the Korean standard pattern identification for stroke (K-SPI-Stroke) questionnaire. *BMC Complement. Altern. Med.* **12**, 55 (2012). <https://doi.org/10.1186/1472-6882-12-55>

28 Kuiwu, Y., Jie, W., Cuiling, Z., Jitao, W. & Juzheng, F. Results of Different Quantitative Diagnosis Analysis on the Symptoms and Signs of Blood Stasis Syndrome in Coronary Heart Disease. *World Science and Technology* **11**, 684-688 (2009). <https://doi.org/10.1016/S1876-3553(10)60032-4>

29 Guan, Y. & He, Q. Liver Cancer: Zheng Classification of Qi Stagnation and Blood Stasis. *Pharmacology & Pharmacy* **05**, 8 (2014). <https://doi.org/10.4236/pp.2014.51012>

30 Tang, X.-d. *et al.* Clinical practice guideline of Chinese medicine for chronic gastritis. *Chin. J. Integr. Med.* **18**, 56-71 (2012). <https://doi.org/10.1007/s11655-012-0960-y>

31 Jung, J. *et al.* Gyejibongneyong-hwan, a herbal medicine for the treatment of dysmenorrhoea with uterine fibroids: a protocol for a randomised controlled trial. *BMJ Open* **6**, e013440 (2016). <https://doi.org/10.1136/bmjopen-2016-013440>

32 Park, Y.-J., Yang, D.-H., Lee, J.-M. & Park, Y.-B. Development of a valid and reliable blood stasis questionnaire and its relationship to heart rate variability. *Complement. Ther. Med.* **21**, 633-640 (2013). <https://doi.org/10.1016/j.ctim.2013.08.019>

33 Han, Y. *et al.* Chinese herbal medicine as maintenance therapy for improving the quality of life for advanced non-small cell lung cancer patients. *Complement. Ther. Med.* **24**, 81-89 (2016). <https://doi.org/10.1016/j.ctim.2015.12.008>

34 Lee, J. A. *et al.* Developing indicators of pattern identification in patients with stroke using traditional Korean medicine. *BMC Res. Notes* **5**, 136 (2012). <https://doi.org/10.1186/1756-0500-5-136>
